# Detection of sleep apnea using smartphone-embedded inertial measurement unit

**DOI:** 10.1038/s41598-025-99801-3

**Published:** 2025-04-28

**Authors:** Junichiro Hayano, Masahiro Takeshima, Aya Imanishi, Masaya Ogasawara, Yasuko Yamada, Emi Yuda, Kazuo Mishima

**Affiliations:** 1 Heart Beat Science Lab, Inc., Sendai, Japan; 2 Nagoya City University, Nagoya, Japan; 3 Department of Neuropsychiatry, Akita University Graduate School of Medicine, Akita City, 010-8543 Japan; 4 Graduate School of Information Sciences, Tohoku University, Sendai, Japan

**Keywords:** Biomarkers, Health care, Biomedical engineering

## Abstract

We previously demonstrated that sleep apnea (SA) can be detected using acceleration and gyroscope signals from smartwatches. This study investigated whether an inertial measurement unit (IMU) embedded in non-wristwatch devices, such as smartphones, can also detect SA when worn during sleep. During polysomnography (PSG), subjects wore an IMU-embedded GPS device (Amue Link^®^) and/or smartphones (Xperia^®^ or iPhone^®^) on their abdomen. Triaxial acceleration and gyroscope signals were recorded overnight. Data were split into training and test groups (2:1) for each device. An algorithm was developed in the training groups to extract respiratory movements (0.13–0.70 Hz) and detect respiratory events, which were validated in the test groups. IMU-derived respiratory events showed breath-by-breath concordance with PSG apnea-hypopnea events, yielding F1 scores of 0.786, 0.821, and 0.796, respectively. Regression model derived from IMU signals correlated with PSG AHI in the test groups (*r* = 0.90, 0.93, and 0.96), with limits of agreement of -16.7 to 25.9, -17.4 to 22.5, and − 18.4 to 20.5. Using cutoff values from the training groups, moderate-to-severe SA (AHI ≥ 15) was identified in the test groups with AUCs of 0.95, 0.98, and 0.94 and F1 scores of 0.89, 0.96, and 0.92, respectively. IMUs embedded in non-wristwatch devices, including smartphones, can quantitatively detect SA when worn during sleep.

**Supplementary Information:**

The online version contains supplementary material available at 10.1038/s41598-025-99801-3.

## Introduction

Sleep apnea (SA) affects an estimated 5–15% of the population^1,2^ and is associated with elevated risks of chronic conditions such as hypertension, diabetes, cardiovascular disease, and dementia. If left untreated, SA can lead to chronic intermittent hypoxia, sympathetic overactivation, systemic inflammation, and endothelial dysfunction, all of which contribute to increased cardiovascular morbidity and mortality^3–6^. Additionally, SA-related excessive daytime sleepiness and cognitive impairment increase the risk of occupational and traffic accidents, further amplifying its societal burden^7,8^.

Although SA is treatable, over 80% of cases remain undiagnosed^9^, posing a substantial burden on healthcare systems worldwide^1,10^. The economic impact of untreated SA, including increased healthcare utilization and productivity loss, is estimated at billions of dollars annually^11^. The low rate of SA diagnosis is partly due to limited access to polysomnography (PSG), the gold standard for diagnosis^12^. PSG requires overnight monitoring in a sleep laboratory, making it costly and time-consuming, with long waiting times in many regions. Home sleep apnea testing (HSAT) offers a more accessible alternative^13–15^. However, many SA patients remain unaware of the need for testing itself unless symptoms are severe, and even simplified screening tools remain underutilized^16^.

A promising approach for improving SA detection is the use of widely available consumer electronics, such as smartwatches and smartphones. The potential of smartwatches as SA screening tools has gained considerable interest^17–22^, with studies reporting strong agreement between smartwatch-detected respiratory events (REs) and PSG-derived apnea-hypopnea index (AHI). Some smartwatch-based methods estimate REs using photoplethysmography (PPG) signals to derive pulse transit time and heart rate variability^18,19,23^, but these methods are susceptibility to motion artifacts and require continuous skin contact. Smartphone applications have also been proposed for SA screening, though many rely on proprietary, unverifiable algorithm^24,25^. Some models utilizing smartphone-recorded breathing sounds have demonstrated potential for detecting obstructive SA^21,26–28^. However, these studies typically provide only whole-night summary statistics without event-by-event analyses, raising concerns that detected events may not directly correspond to PSG-scored respiratory events^29^.

In a previous study, we introduced a novel approach using an inertial measurement unit (IMU) embedded in a smartwatch to detect SA-related REs through acceleration and gyroscope signals generated by subtle respiratory-related arm movements^30,31^ IMU technology offers several advantages, including low power consumption, indirect monitoring without requiring skin contact, and widespread availability in wearable devices.

The present study expands on the prior findings by evaluating whether IMUs embedded in non-wristwatch portable devices can also detect SA. We developed an algorithm to analyze IMU signals from commercially available Android^®^ and iOS^®^ smartphones, as well as a global positioning system (GPS) device worn on the abdomen during sleep. The algorithm’s performance was assessed in two key ways:

1. breath-by-breath concordance with apnea-hypopnea events detected by standard PSG.
2. Correlation between IMU-detected RE frequency and PSG-derived AHI.

By validating IMU-based SA detection across multiple devices and evaluation metrics, this study aims to establish it as a versatile, cost-effective, and scalable screening solution utilizing readily available consumer electronics.

## Methods

### Ethics approval and consent to participate

All procedures were performed in accordance with the Regulations Concerning the Conduct of Life Science and Medical Research Involving Human Subjects at Tohoku University, Japan, the Ethical Guidelines for Medical Research Involving Human Subjects issued by the Japanese Ministry of Health, Labor and Welfare, and the 1964 Declaration of Helsinki and its subsequent amendments. The study protocol was approved by the Ethics Committee of Tohoku University Hospital, Sendai, Japan (registration number 34220, approval date: 2023/12/28). All subjects provided written informed consent to participate in the study.

### Subjects

The study included consecutive, eligible patients who underwent overnight PSG from January 2024 to April 2024 at Akita University Hospital (Akita City, Japan), as well as sleep clinics of Medical Corporation Sound Sleep (Japan) and Medical Corporation Zuimeikai (both in Japan) for the diagnosis of SA. The inclusion criterion was adults aged 20–80 years who provided written informed consent to participate voluntarily. Exclusion criteria included acute or chronic illness requiring hospitalization within the past three months, a history of skin allergies to respiration-sensing bands, inability to provide informed consent, and pregnancy or possible pregnancy.

### Protocol

**Fig. 1:**
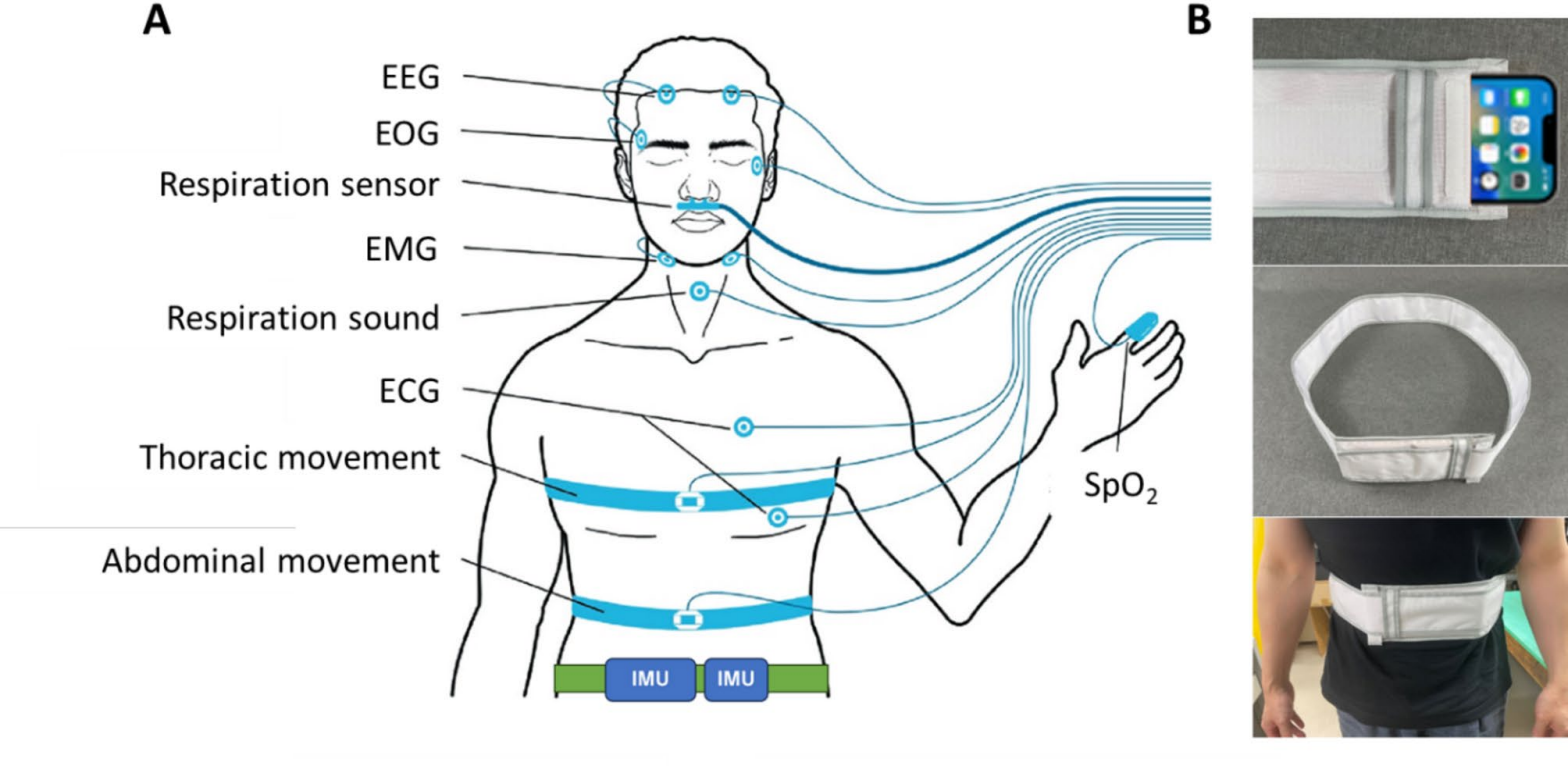
Positions for attaching the sensors for polysomnography and inertial-measurement-unit (IMU)-embedded devices (**A**). The IMU devices were held on the lower abdomen with a 10 cm wide polyester belt containing pockets to prevent movement or rotation during measurement (**B**). The belt was wrapped around the waist with a secure, comfortable fit that did not impede breathing and was fastened with Velcro. *EEG* electroencephalogram, *EOG* electrooculogram, *EMG* electromyogram, *ECG* electrocardiogram.

Subjects arrived at the participating sleep laboratory in the evening and stayed overnight in a PSG testing chamber. During the PSG, IMU devices were positioned on the lower abdomen in a way that did not interfere with polysomnography measurements. Each device was secured in place using a 10 cm wide polyester belt with pockets to prevent movement or rotation during the measurements. The belt was comfortably wrapped around the waist and fastened with Velcro, ensuring it did not impede breathing (Fig. 1).

We examined three IMU-embedded devices: a GPS device (Amue Link^®^ LM-001, Sony Network Communications Inc., Tokyo, Japan), an Android smartphone (Xperia 8^®^ Lite, Sony Corporation, Tokyo, Japan), and an iOS smartphone (iPhone SE^®^ 3rd generation, iOS 17.2.1, Apple Inc., Cupertino, CA, USA). These devices are referred to as Amue Link, Xperia, and iPhone, respectively. Two devices, randomly selected from Amue Link, Xperia, and iPhone, were attached to each subject. However, due to limited device availability in each sleep laboratory, only one device was used for some subjects, and the combination of Xperia and iPhone was not included.

PSG recordings were analyzed offline using sleep diagnostic software (Remlogic version 3.4.1, Natus Medical Incorporated, Middleton, Wisconsin, USA; DOMINO Ver. 3.0.0.6, SOMNO medics, Coral Gables, Florida, USA). Automated analysis results were reviewed and edited by expert sleep technicians (Certified Sleep Medicine Examiners by the Japan Sleep Society). The acceleration and gyroscope signals from Amue Link were temporarily stored in a buffer and transferred via LTE-M communication to a secure cloud storage prepared for this study. Signals from smartphones were stored in memory and transferred via mobile data or Wi-Fi, depending on the sleep laboratory environment.

For each device, subjects were randomly divided into training and test groups in a 2:1 ratio, ensuring balanced AHI distribution between the groups. In the training groups, an algorithm was developed to detect respiratory events (REs) from IMU signals, and models were created to estimate the AHI based on the frequency of REs in the IMU signals. The models were then evaluated using the test groups.

### Measurements

PSG was recorded overnight using the standard PSG montages: F4-M1, F3-M2, C4-M1, C3-M2, O2-M1, O1-M2 electroencephalograms, left and right electrooculograms, submental electromyogram (EMG), nasal pressure cannula, oronasal airflow, left and right tibial EMGs, thoracoabdominal inductance plethysmograms, pulse oximetric arterial blood oxygen saturation (SpO2), a neck microphone, body position sensors, and a modified lead II ECG.

Respiratory events were scored according to the American Association of Sleep Medicine (AASM) Manual for the Scoring of Sleep and Associated Events, Version 2.5. The average hourly frequencies of apnea episodes, hypopnea episodes, and their combination were defined as the apnea index, hypopnea index, and AHI, respectively. The frequencies of different types of apnea (obstructive, central, and mixed) were also recorded. Subjects with an AHI of < 5, 5–15, 15–30, and ≥ 30 were classified as having no SA, mild SA, moderate SA, and severe SA, respectively. Body positions were detected by the body position sensor and recorded at each postural change.

The IMU devices recorded acceleration and gyroscope signals at sampling frequencies of 32 Hz (Amue Link) and 30 Hz (Xperia, iPhone). The resolution of the acceleration signal was 0.061 mG per least significant bit (± 2.0 G at 16-bit), and the gyroscope resolution was 0.0076 degrees per second (dps) per least significant bit (± 250 dps at 16-bit). Acceleration and gyroscope data were obtained from Android and iOS smartphones using the standard system functions, *SensorManager* (Android Developers, Google LLC) and *CMMotionManager* (Apple Developer, Apple Inc), respectively.

### Data analysis

**Fig. 2:**
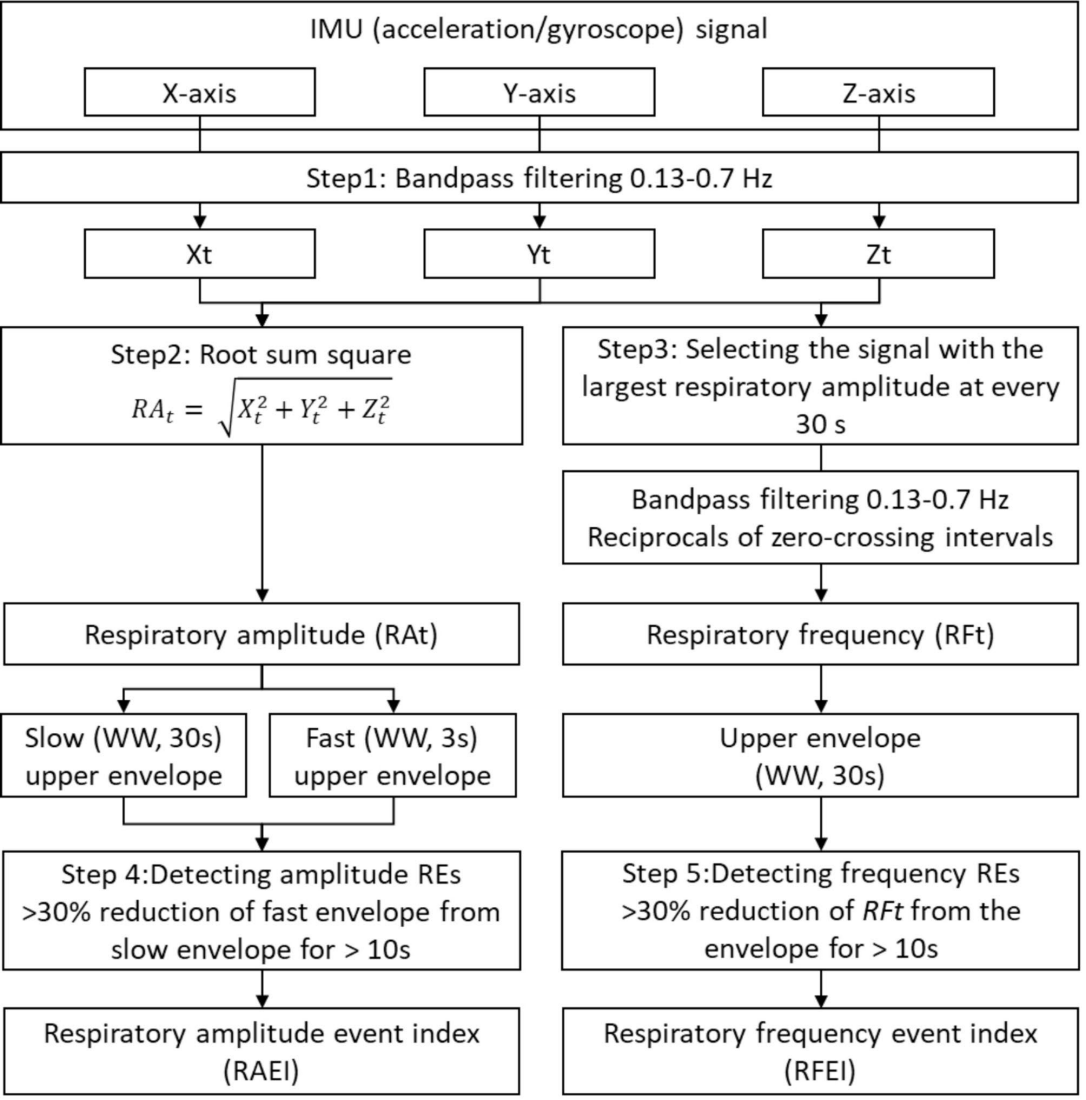
Flowchart of data analysis for detecting respiratory events (Res) in the IMU signals. The same algorithm was used for acceleration and gyroscope signals and calculated respiratory amplitude event index (RAEI) and respiratory frequency event index (RFEI) for each signal.

Figure 2 shows the flowchart of the data analysis process. Respiratory events (REs) were detected from both acceleration and gyroscope signals by the same algorithm but separately. The algorithm consisted of the following five steps.

#### Step 1: extraction of the respiratory components

The X-, Y-, and Z-axis signals were processed individually using a finite impulse response (FIR) band-pass filter (0.13–0.7 Hz) to extract the respiratory components in each axis. This filter was applied to exclude non-respiratory components such as pulse waves (0.7–2.5 Hz), coarse body movements (2–4 Hz), and seismocardiogram/gyrocardiogram signals (≥ 8 Hz) )^32^.

Here, instead of applying the band-pass filter to a combined signal of the X-, Y-, and Z-axes, the filter was applied individually to each axis to enable effective extraction of the respiratory waveform in Step 3 (see Appendix Figure S1). The frequency range (0.13–0.7 Hz) was selected based on a prior study^30^ to ensure that the amplitude of the filtered respiratory wave is preserved when respiratory frequency is kept > 0.13 Hz (cycle length < 7.7 s) and is suppressed when respiration ceases for > 7.7 s (including SA events lasting > 10 s) (see Appendix Figure S2).

#### Step 2: measurement of respiratory amplitude

The band-pass filtered time series of the X-, Y-, and Z-axes were combined into a single scalar that reflects the amplitude of respiratory movement (RAt), calculated as:

$$\:{RA}_{t}=\:\sqrt{{X}_{t}^{2}+{Y}_{t}^{2}+{Z}_{t}^{2}}$$

#### Step 3: measurement of respiratory frequency

Of the band-pass filtered X-, Y-, and Z-axis time series obtained in Step 1, the one that most strongly reflected respiratory movement (i.e., the one with the largest excursion range) was selected every 30 s. The selected time series (each 30 s in length) were then concatenated to form a single respiratory waveform time series, with polarity adjustments ensuring phase continuity at the junctions. This method effectively tracks respiratory waveforms even when the orientation of the respiratory movement vector within the IMU’s X-, Y-, and Z-axis system changes due to postural changes during sleep (see Appendix Figure S2).

After the concatenated signal was passed through the same band-pass filter (0.13–0.7 Hz) to prevent artifacts at the connection points, respiratory cycle lengths were estimated by detecting the intervals between consecutive zero-crossing points from negative to positive in the respiratory waveform time series. The time series of respiratory cycle lengths was then interpolated using a step function, where the instantaneous cycle length at a given time point was assumed to be the cycle length associated with that point. Finally, the respiratory frequency time series (RFt) was obtained as the reciprocal of the interpolated cycle lengths, sampled at equal intervals.

#### Step 4: detection of REs from respiratory amplitude

Following the AASM Manual for the Scoring of Sleep and Associated Events, which defines sleep apnea and hypopnea as events with a reduction in respiratory amplitude of > 90% and > 30%, respectively, for more than 10 s, our approach identifies an RE when the respiratory amplitude (RAt) decreases by > 30% for > 10 s.

**Fig. 3:**
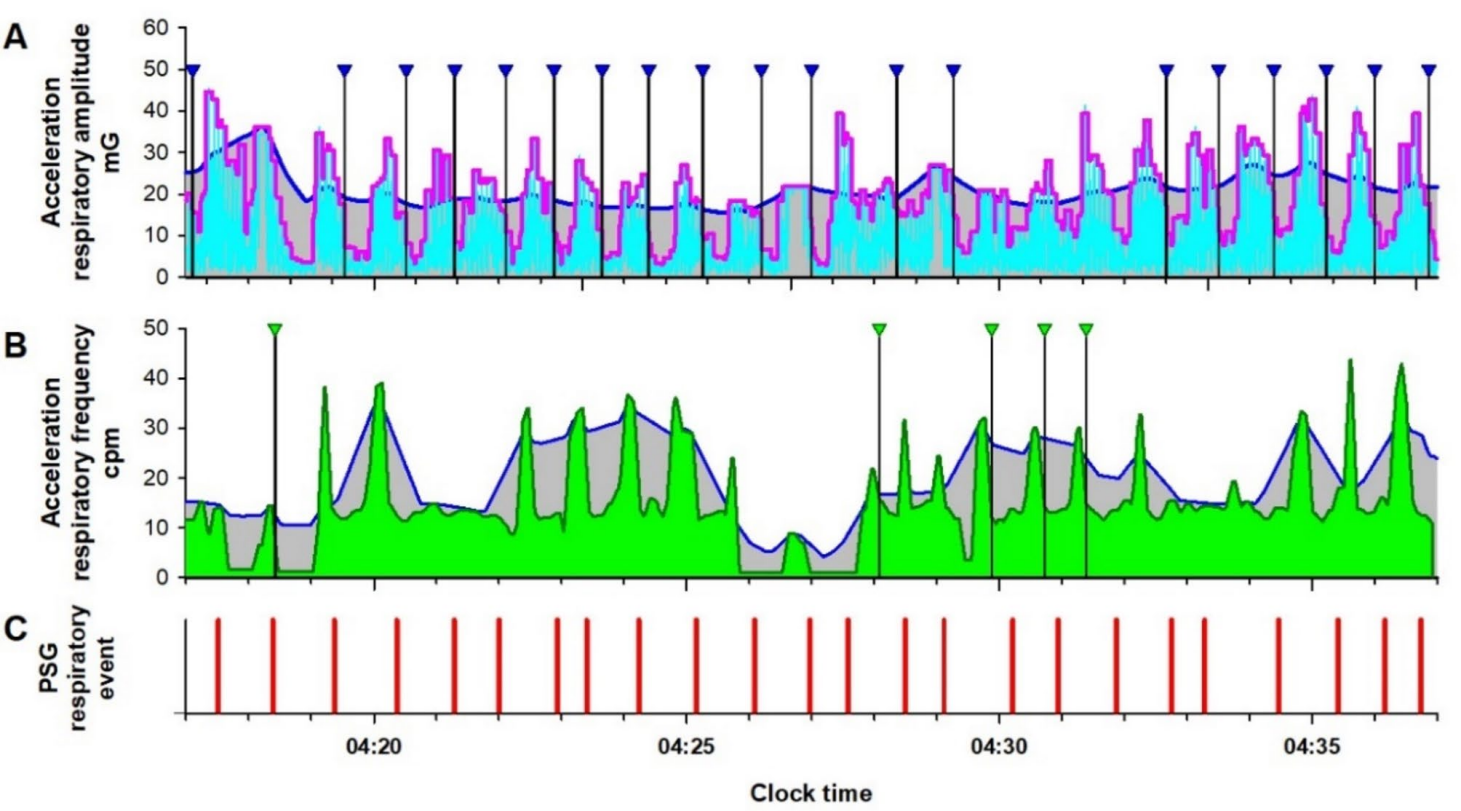
Respiratory amplitude (cyan line in (**A**)) and frequency (dark green line in (**B**)) extracted from IMU acceleration signal of an Android smartphone in a representative subject with sever sleep apnea during a PSG examination. In (**A**), the magenta and blue lines are the fast and slow envelopes (95th percentiles within 3-s and 30-s moving windows) of the respiratory amplitude, respectively. The black vertical lines with blue triangles are respiratory events (REs) detected as a > 30% reduction in the fast envelope from the slow envelope lasting 10–90 s. In (**B**), blue line is the upper envelope (95th percentile of 30-s moving window) of the respiratory frequency. Vertical black bars with green triangles are REs detected as > 30% reduction in the respiratory frequency from the envelope lasting 10–90 s. To avoid double counting of RE by both respiratory amplitude and frequency, the RE detection by the respiratory frequency was suppressed while the fast envelope of the respiratory amplitude was > 30% below the slow envelope. In (**C**), vertical red bars are RE (apnea and hypopnea episodes) detected by the simultaneous PSG.

**Fig. 4:**
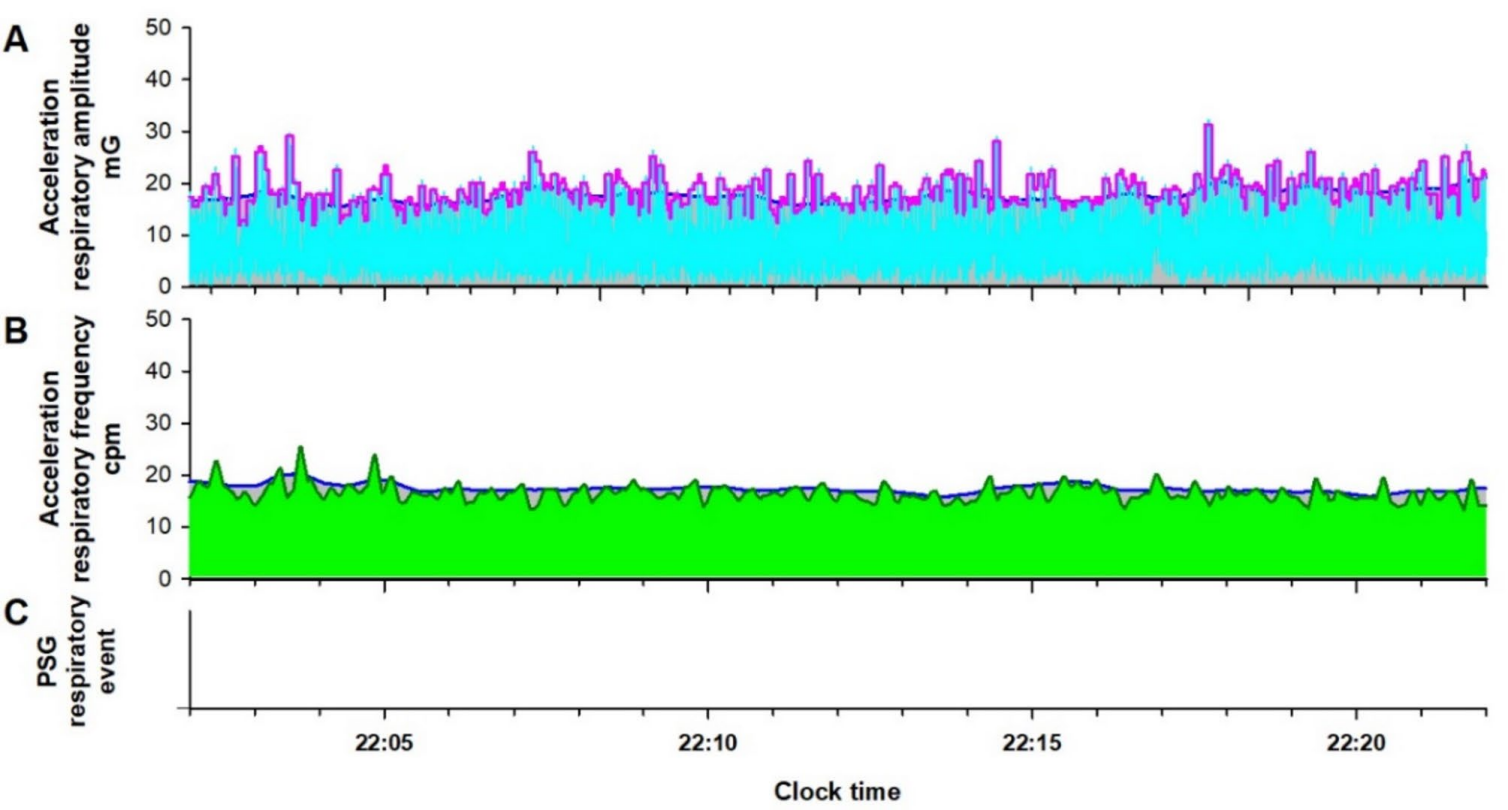
Respiratory amplitude (cyan line in (**A**)) and frequency (dark green line in (**B**)) extracted from the IMU acceleration signal of an Android smartphone during normal breathing in a subject undergoing a PSG examination. In (**A**), the magenta and blue lines, which show the fast and slow envelopes of the respiratory amplitude, respectively, overlap, and RE is not detected. (**B**) The blue line, which shows the upper envelope of the respiratory frequency, overlaps with the respiratory frequency itself, and RE is also not detected. RE is not detected in the simultaneous PSG (**C**).

For the time series of estimated respiratory amplitude (RAt) (cyan lines in Figs. 3A and 4A), moving averages of the envelope (95th percentile values) were calculated using window widths of 3 and 30 s, representing the fast and slow envelopes, respectively (magenta and blue lines in Figs. 3A and 4A). The fast envelope was assumed to reflect breath-by-breath variations in respiratory amplitude, while the slow envelope represented the baseline amplitude, smoothing out SA-induced changes. A > 30% reduction in the fast envelope relative to the slow envelope, persisting for 10 to 90 s, was identified as an RE (vertical black line with blue triangle in Fig. 3A).

The 95th percentile, rather than the maximum value, was used to calculate the envelopes to minimize artifacts caused by transient noise. Additionally, limiting the RE duration to a maximum of 90 s helped prevent the misclassification of prolonged signal loss as an RE.

#### Step 5: complementary detection of REs from respiratory frequency

For the time series of estimated respiratory frequency (RFt) (dark green lines in Figs. 3B and 4B), the upper envelope was calculated as the 95th percentile within a 30-second window (blue lines in Figs. 3B and 4B). A > 30% reduction in RFt from this envelope, persisting for 10 to 90 s, was identified as an RE (vertical black line with green triangle in Fig. 3B). To prevent double counting of REs detected by both RAt and RFt, RE detection based on RFt was suppressed when the fast envelope of RAt was > 30% below the slow envelope.

As shown in Fig. 4, during normal breathing, the fast and slow envelopes of RAt, as well as the upper envelope of RFt and RFt itself, overlap, preventing the detection of REs. The number of REs detected from RAt and RFt was divided by the monitoring time to calculate the respiratory amplitude event index (RAEI) and the respiratory frequency event index (RFEI), respectively.

### Breath-by-breath concordance between REs detected by IMU and apnea-hypopnea events by PSG

To assess the temporal concordance between REs detected from IMU signals and apnea-hypopnea events detected by PSG, both were mapped onto a breath-by-breath time axis. The entire time axis was segmented into consecutive respiratory periods based on the respiratory nadir points of the abdominal inductance plethysmograms. Respiratory periods containing at least one RE, detected from either RAt or RFt of acceleration or gyroscope signals, were labeled as RE-positive breaths, while all others were labeled as RE-negative breaths. Similarly, respiratory periods were classified as apnea-hypopnea-positive or -negative based on PSG-detected apnea-hypopnea events. Concordance was evaluated in both the training and test groups for each device and body position, allowing for a one-breath gap to account for variations in event flag placements between the IMU and PSG analysis programs, which may mark an event at any point from onset to termination.

### Creation and validation of models to estimate AHI

To evaluate whether RAEI and RFEI from IMU signals can predict SA severity, we developed a multiple regression model to estimate the RE index (REI), with PSG-derived AHI as the dependent variable and RAEI and RFEI from acceleration and gyroscope signals as independent variables. Separate regression models were trained for each device using the training groups and evaluated in the test groups.

For each device, the optimal REI cutoffs for detecting moderate-to-severe SA (PSG AHI ≥ 15) and severe SA (AHI ≥ 30) were determined using receiver operating characteristic (ROC) analysis in the training groups. The classification performance of these cutoffs was then validated in the test groups using sensitivity, specificity, and the F1 score.

### Statistical analysis

Statistical analyses were performed using the Statistical Analysis System (SAS, SAS Institute, Cary, NC, USA). Differences in quantitative and categorical variables between the training and test groups were assessed using the Wilcoxon rank sum test and the χ² test, respectively. Paired t-tests were used to compare values derived from acceleration and gyroscope signals within individual subjects, and their relationships were evaluated using Pearson’s correlation coefficient and root mean squared error (RMSE).

Breath-by-breath concordance between REs detected by IMU signals and apnea-hypopnea events detected by PSG was assessed in both the training and test groups using sensitivity, specificity, accuracy, positive predictive value (PPV), negative predictive value (NPV), and the F1 score.

Multiple regression models were developed using the REG procedure to predict PSG-derived AHI based on RAEI and RFEI from acceleration and gyroscope signals. Model performance was evaluated using Pearson’s correlation coefficient, RMSE, and Bland-Altman analysis comparing PSG AHI with REI (the predicted AHI).

To assess REI’s ability to classify SA severity, ROC curve analysis was conducted, with the area under the curve (AUC) as a measure of classification performance. The optimal REI cutoffs for each SA severity level were determined in the training groups and then validated in the test groups using sensitivity, specificity, accuracy, PPV, NPV, and the F1 score. Statistical significance was set at *P* < 0.05.

## Results

### Subjects’ characteristics

**Fig. 5:**
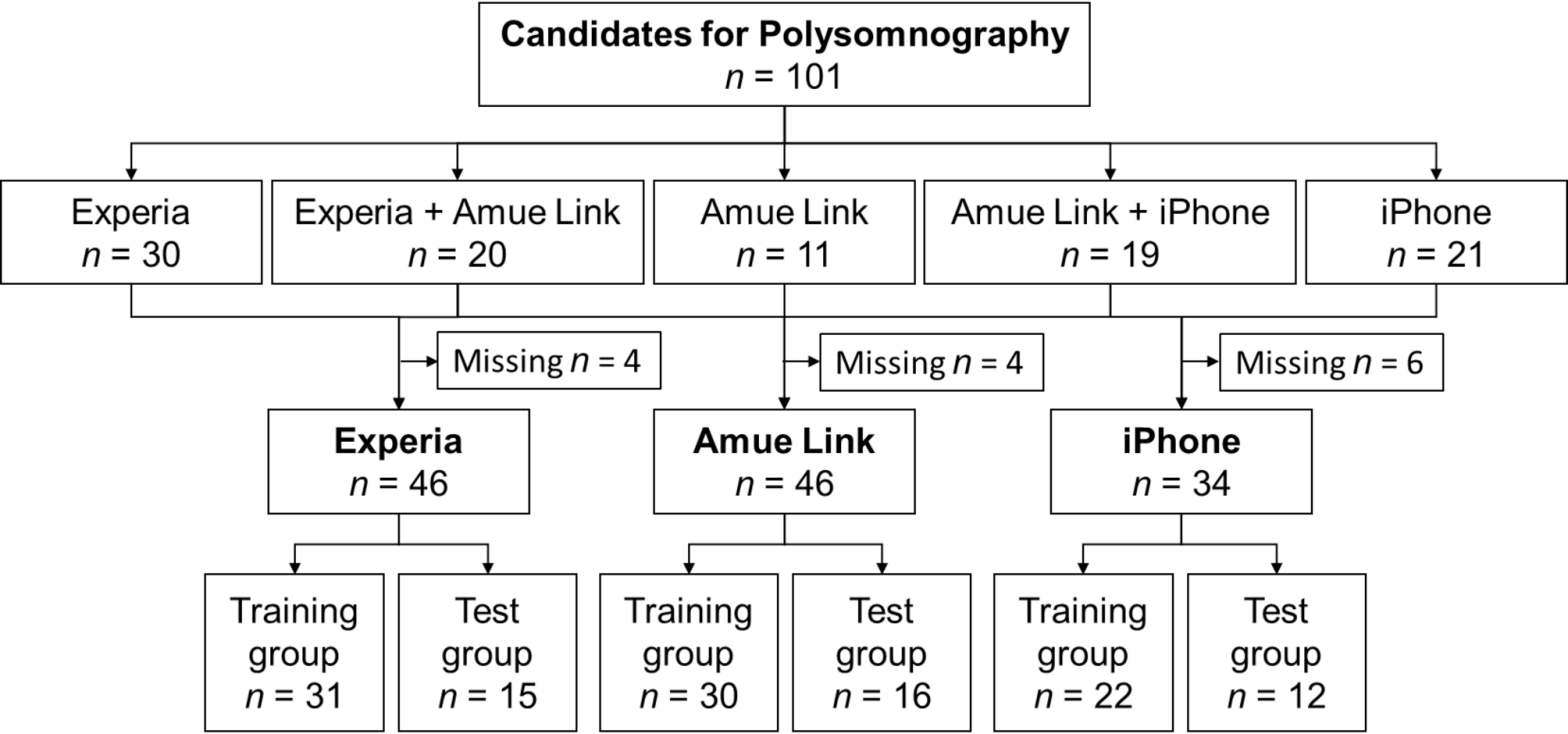
Research subject selection diagram.

Data were obtained from 46 subjects for both Amue Link and Xperia, and 36 subjects for iPhone. Due to data transmission errors, recordings were lost for 4 subjects using Amue Link, 4 subjects using Xperia, and 6 subjects using iPhone (Fig. 5). The final dataset included 42 subjects for Amue Link, 42 for Xperia, and 30 for iPhone.

The characteristics of subjects measured by each device are summarized in Table 1. Participants were randomly divided into training and test groups for each device. No significant differences were observed in baseline characteristics between the two groups (Appendix Table S1).

**Table 1:** Characteristics of subjects for each device.

	Amue link	Xperia	iPhone
Number of subjects	46	46	34
Age (years)	45 (33–56)	48 (42–62)	45 (33–53)
Female, *n* (%)	6 (14%)	4 (9%)	9 (26%)
BMI (kg/m^2^)	26.2 (23.2–30.4)	26.6 (23.3–30.4)	24.6 (21.6–27.3)
Polysomnography			
TRT (min)	512 (470–530)	528 (494–553)	522 (487–549)
TST (min)	404 (337–438)	401 (346–431)	387 (303–444)
Sleep efficiency (%)	84.5 (78.2–87.5)	82.5 (76.5–89.3)	85.4 (78.6–92.0)
Sleep latency (min)	33.3 (16.0–55.5)	27.8 (13.0–61.0)	25.0 (10.5–50.0)
WASO (min)	28.5 (21.5–45.0)	40.8 (27.5–59.5)	36.5 (25.0–54.5)
Sleep stage			
N1 (%)	21 (14–35)	25 (14–37)	20 (13–32)
N2 (%)	50 (39–59)	49 (39–56)	52 (43–61)
N3 (%)	12 (5–21)	7 (3–18)	12 (4–21)
REM (%)	11 (4–19)	11 (5–20)	9 (1–19)
Respiratory rate (cpm)	14.1 (12.9–16.1)	14.6 (13.8–16.5)	14.5 (13.1–15.4)
AHI	15.2 (8.5–35.3)	24.0 (10.4–45.0)	12.01(5.0–35.0)
OAI	3.5 (1.2–7.2)	6.6 (1.2–17.7)	3.2 (0.7–6.1)
CAI	0.4 (0.2–1.8)	0.9 (0.2–3.6)	0.6 (0.2–1.6)
MAI	0.2 (0.1–0.8)	0.4 (0.1–2.3)	0.4 (0.0–1.1)
HI	9.8 (4.0–19.2)	12.1 (7.1–20.4)	8.8 (3.2–15.1)
AHI			
< 5	8 (17%)	5 (22%)	9 (26%)
5–15	14 (30%)	10 (22%)	9 (26%)
15–30	9 (20%)	13 (28%)	6 (18%)
≥ 30	15 (33%)	18 (38%)	10 (30%)

Data are median (IQR) or frequency (%).

*AHI* apnea-hypopnea index, *BMI* body mass index, *CAI* central apnea index, *HI* hypopnea index, *MAI* mixed apnea index, *OAI* obstructive apnea index, *TRT* total recording time, *TST* total sleep time, *WASO* wake after sleep onset.

### Detection of REs and their breath-by-breath concordance with PSG apnea-hypopnea events

Analysis of data from the Amue Link, Xperia, and iPhone training groups confirmed that the algorithm described in the Data Analysis section could be applied to both acceleration and gyroscope signals from all three devices to detect REs and their temporal positions.

Breath-by-breath concordance was assessed by mapping IMU-detected REs and PSG-detected apnea-hypopnea events onto the time axis. In the training groups, RE-positive breathes detected apnea-hypopnea-positive events with sensitivities of 70.1% (Amue Link), 69.2% (Xperia), and 65.6% (iPhone), and PPVs of 83.8%, 88.7%, and 75.7%, respectively (Table 2). This performance was validated in the test groups, yielding sensitivities of 77.5%, 78.8%, and 74.7%, and PPVs of 79.8%, 85.6%, and 85.2%, respectively.

**Table 2:** Breath-by-breath classification performance for each device in the training and test groups. Each breath was labeled as being either apnea/hypopnea or normal breathing based on polysomnographic judgment and was classified as positive or negative based on respiratory events (REs) detected as a reduction in respiratory amplitude or frequency, derived from acceleration and gyroscope signals.

Device	Group	Number of breathes				Classification performance				
		TP	FP	FN	TN	Sensitivity	Specificity	PPV	NPV	F1 score
Amue link	Training	2915	564	1242	183,343	70.1%	99.7%	83.8%	99.3%	0.763
	Test	1809	459	524	98,715	77.5%	99.5%	79.8%	99.5%	0.786
Xperia	Training	3970	504	1769	205,783	69.2%	99.8%	88.7%	99.1%	0.777
	Test	2359	398	634	109,034	78.8%	99.6%	85.6%	99.4%	0.821
iPhone	Training	1764	565	924	146,349	65.6%	99.6%	75.7%	99.4%	0.703
	Test	1281	223	433	75,765	74.7%	99.7%	85.2%	99.4%	0.796

*TP* true positive, *FP* false positive, *FN* false negative, *TN* true negative, *PPV* positive predictive value, *NPV* negative predictive value.

**Fig. 6:**
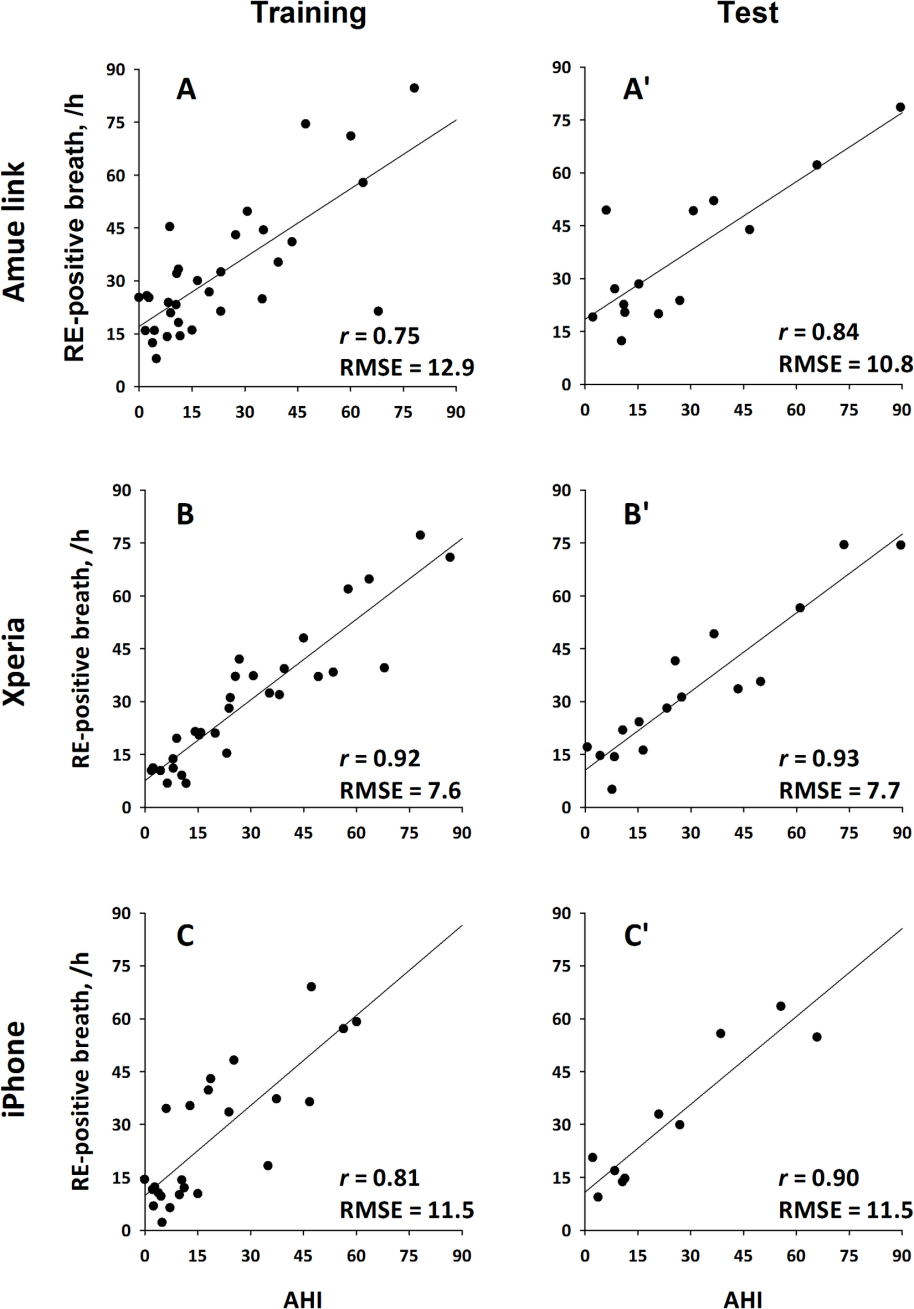
Relationships between apnea-hypopnea index (AHI) of polysomnography and the hourly frequency of RE-positive breaths obtained from smartphone IMU signals in the training (**A**–**C**) and test groups (**A**ʹ–**C**ʹ); (**A**, **A**ʹ) Amue Link, (**B**, **B**ʹ) Xperia, and (**C**, **C**ʹ) iPhone. *r* correlation coefficient, *RE* respiratory event, *RMSE* root mean squared error.

Additionally, the hourly frequency of RE-positive breaths strongly correlated with PSG-derived AHI in the training groups, with correlation coefficients (*r*) of 0.75 (Amue Link), 0.92 (Xperia), and 0.81 (iPhone). These correlations were further confirmed in the test groups (*r* = 0.84, 0.93, and 0.90) (Fig. 6).

### Regression-based estimation of AHI

The algorithm calculated hourly RE frequencies from respiratory amplitude and frequency signals, termed RAEI and RFEI, respectively, for both acceleration and gyroscope data in individual subjects. Although RAEI from acceleration signal was lower than that from the gyroscope signal, the two were closely correlated across all three devices (Table 3). In contrast, RFEI values did not differ significantly between acceleration and gyroscope signals, though their correlation strength varied among devices.

**Table 3:** Comparison of hourly frequencies of respiratory events (RE) observed in the respiratory amplitude and respiratory frequency between the acceleration and gyroscope signals in the training groups of each device.

	Acceleration	Gyroscope	P*	*r*	RMSE
RAEI					
Amue link	10.9 ± 8.3	12.5 ± 10.4	0.03	0.93	3.04
Xperia	13.9 ± 11.0	18.0 ± 13.9	< 0.0001	0.98	2.06
iPhone	11.6 ± 9.5	14.8 ± 13.0	0.002	0.98	2.03
RFEI					
Amue link	11.3 ± 8.7	12.5 ± 8.6	0.3	0.65	6.72
Xperia	11.3 ± 8.3	11.1 ± 7.8	0.6	0.90	3.58
iPhone	11.2 ± 7.6	11.6 ± 6.2	0.6	0.85	4.10

*RAEI* hourly frequency of respiratory event detected from respiratory amplitude, *RFEI* hourly frequency of respiratory event detected from respiratory frequency, *r* correlation coefficient between values from acceleration and gyroscope signals, *RMSE* root mean squared error.

*Significance of difference of paired t-test between values from acceleration and gyroscope signals.

The relationships between RAEI, RFEI, and PSG-derived AHI differed across devices. Therefore, multiple regression models were developed separately for each device to estimate PSG AHI using RAEI and RFEI from both acceleration and gyroscope signals in their respective training groups.

**Fig. 7:**
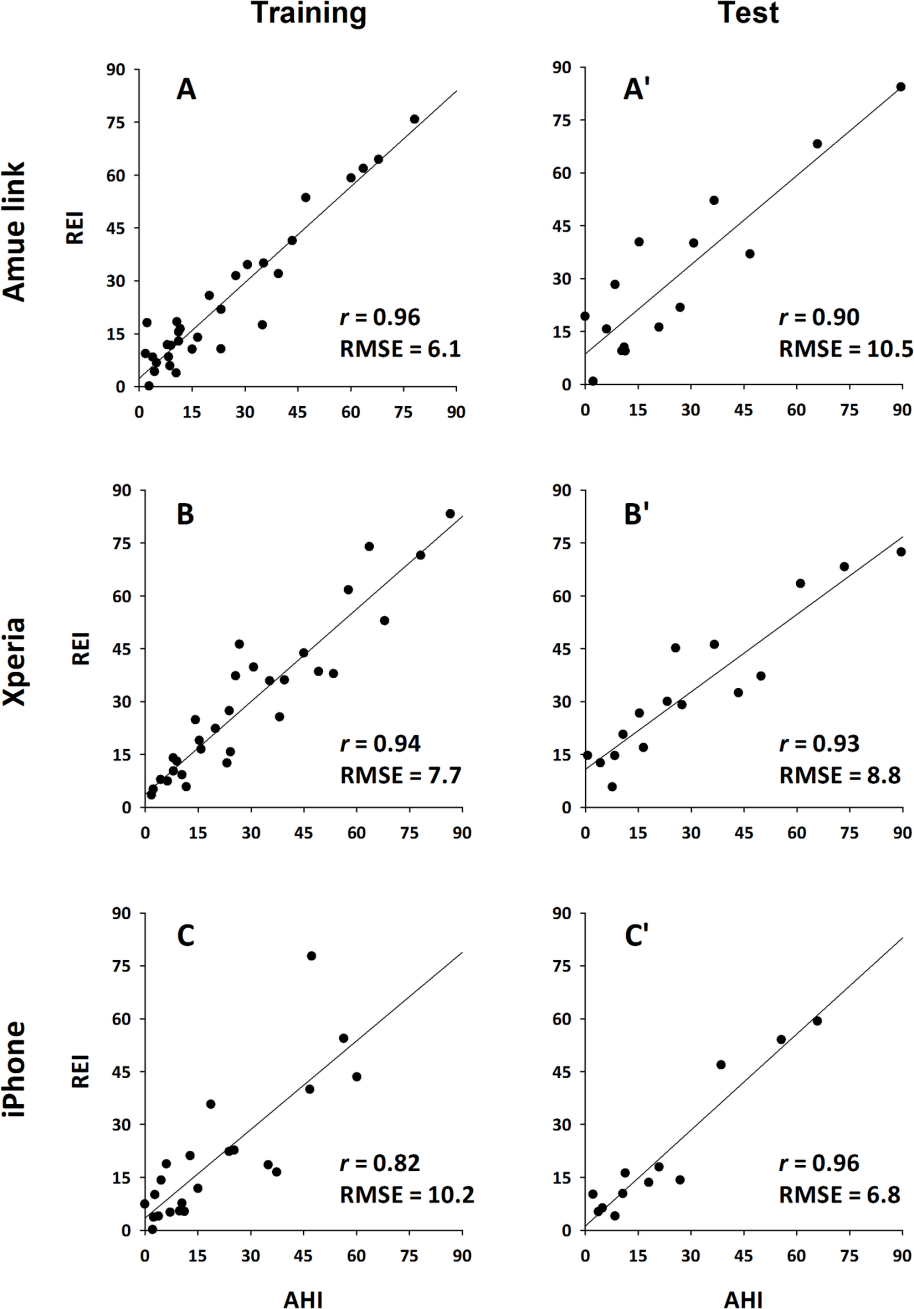
Relationships between apnea-hypopnea index (AHI) of polysomnography and respiratory event index (REI) obtained from IMU signals in the training (**A**–**C**) and test groups (**A**ʹ–**C**ʹ) of each device; (**A**, **A**ʹ) Amue Link, (**B**, **B**ʹ) Xperia, and (**C**, **C**ʹ) iPhone. The abbreviations are provided in the legend of Fig. 6.

Table 4 presents the multiple regression coefficients for predicting PSG AHI in the training groups of each device. The relationships between PSG AHI and regression-derived REI in the training groups are shown in the left-side panels of Fig. 7. Across all devices, REI exhibited a strong correlation with PSG AHI (*r* = 0.96, 0.94, and 0.84 for Amue Link, Xperia, and iPhone, respectively). This strong correlation was also confirmed in the test groups, where the regression-derived REI remained highly correlated with PSG AHI (*r* = 0.90, 0.93, and 0.96, respectively) (right-side panels of Fig. 7). The fact that the test group correlations were not substantially lower than those in the training groups suggests the models were not overfitted.

**Table 4:** AHI regression model coefficients of hourly frequencies of respiratory events (RE) obtained from the respiratory amplitude and frequency of the acceleration and gyroscope signals in the training groups of each device.

	Regression coefficient				
	Acceleration		Gyroscope		Intercept
	RAEI	RFEI	RAEI	RFEI	
Amue Link	1.71	0.20	0.82	− 0.44	− 2.60
Xperia	1.43	0.17	0.84	0.21	− 5.64
iPhone	0.34	0.95	0.71	− 0.62	1.36

Abbreviations are defined in the footnote to Table 2.

**Fig. 8:**
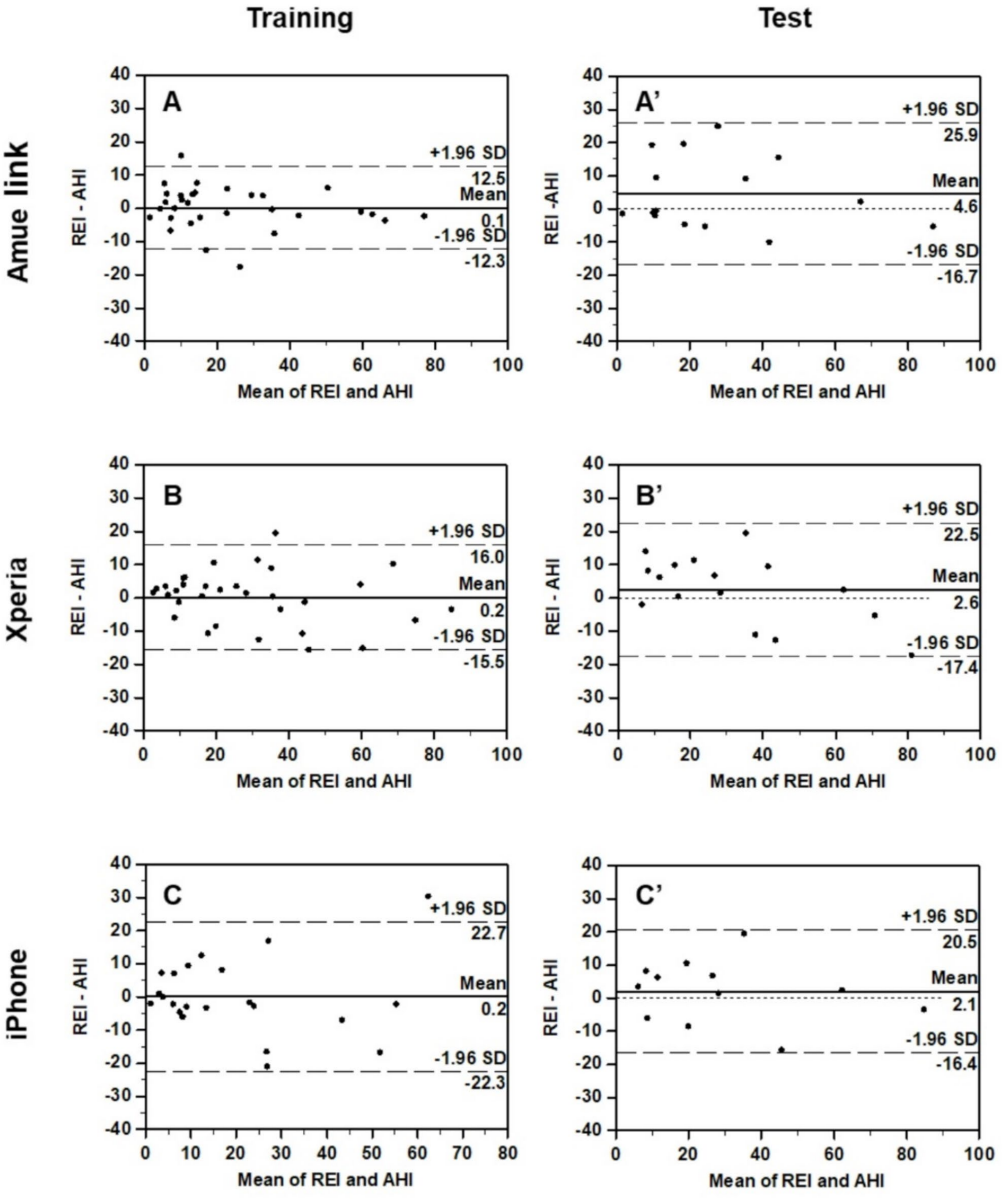
Bland-Altman plots between polysomnographic AHI and smartphone-IMU REIs in the training (**A**–**C**) and test groups (**A**ʹ–**C**ʹ); (**A**, **A**ʹ) Amue Link, (**B**, **B**ʹ) Xperia, and (**C**, **C**ʹ) iPhone. Horizontal dotted lines indicate mean difference and dashed lines indicate the limits of agreement (± 1.96 SD) between two measures.

Figure 8 presents Bland-Altman plots illustrating the agreement between REI and PSG AHI in both training and test groups for each device. In the training groups, the mean differences were 0.1, 0.2, and 0.2, with limits of agreement of -12.3 to 12.5, -15.5 to 16.0, and − 22.3 to 22.7 for Amue Link, Xperia, and iPhone, respectively (left-side panels of Fig. 8). In the test groups, mean differences were slightly larger (4.6, 2.6, and 2.1), with limits of agreement of -16.7 to 25.9, -17.4 to 22.5, and − 16.4 to 20.5, respectively (right-side panels of Fig. 8).

### Estimation of SA severity

In the training groups, the REIs obtained from the models for Amue Link, Xperia, and iPhone effectively discriminated subjects with moderate-to-severe SA (AHI ≥ 15), achieving areas under the ROC curve (AUCs) of 0.92, 0.97, and 0.94 and F1 scores of 0.84, 0.95, and 0.82, respectively (Table 5). When the cutoff values derived in the training groups were applied to the test groups, subjects with moderate-to-severe SA were classified with F1 scores of 0.89, 0.96, and 0.92, respectively.

**Table 5:** Classification performance of each device for moderate-to-severe sleep apnea (AHI ≥ 15) by REI in the training and test groups.

Device	Group	AUC (SE)	REI cutoff*	Sensitivity	Specificity	PPV	NPV	F1 score
Amue link	Training	0.92 (0.049)	≥ 17	81%	87%	87%	81%	0.84
	Test	0.95 (0.053)		100%	71%	80%	100%	0.89
Xperia	Training	0.97 (0.030)	≥ 15	95%	90%	95%	90%	0.95
	Test	0.98 (0.026)		100%	80%	92%	100%	0.96
iPhone	Training	0.94 (0.045)	≥ 13	90%	75%	75%	90%	0.82
	Test	0.94 (0.066)		100%	83%	86%	100%	0.92

*Cutoff values for REI were determined for each device in the training groups and applied to the test groups for that device.

Similarly, the models for Amue Link, Xperia, and iPhone discriminated subjects with severe SA (AHI ≥ 30) with AUCs of 0.98, 0.95, and 0.90 and F1 scores of 0.90, 0.88, and 0.80 in the training groups (Table 6). When applying the same cutoff values to the test groups, classification performance remained high, with AUCs of 0.96, 0.97, and 1.00 and F1 scores of 0.91, 0.92, and 1.00, respectively.

**Table 6:** Classification performance of each device for severe sleep apnea (AHI ≥ 30) by REI in the training and test groups.

Device	Group	AUC (SE)	REI cutoff*	Sensitivity	Specificity	PPV	NPV	F1 score
Amue link	Training	0.98 (0.026)	≥ 30	90%	95%	90%	95%	0.90
	Test	0.96 (0.047)		100%	90%	83%	100%	0.91
Xperia	Training	0.95 (0.038)	≥ 30	92%	89%	85%	94%	0.88
	Test	0.97 (0.039)		100%	90%	86%	100%	0.92
iPhone	Training	0.90 (0.077)	≥ 36	67%	100%	100%	89%	0.80
	Test	1.00 (0.000)		100%	100%	100%	100%	1.00

*Cutoff values for REI were determined for each device in the training groups and applied to the test groups for that device.

To further assess classification performance, SA severity was categorized into four levels: no SA, mild SA, moderate SA, and severe SA (Table 7). The cutoff values for REI to estimate each severity level were determined in the training groups and validated in the test groups for each device. The macro F1 scores (mean of F1 scores across all severity classes) were 0.586, 0.699, and 0.646 in the training groups and 0.677, 0.788, and 0.714 in the test groups for Amue Link, Xperia, and iPhone, respectively.

**Table 7:** Confusion matrix of each device for classification of PSG SA severity by REI in the training and test groups.

Ame Link	Training group				Test group			
	PSG				PSG			
	No SA	Mild	Moderate	Severe	No SA	Mild	Moderate	Severe
REI < 5	**2**	1	0	0	**1**	0	0	0
5–17	3	**7**	3	0	0	**4**	1	0
17–30	1	1	**2**	1	1	1	**1**	0
REI > 30	0	0	1	**9**	0	0	1	**5**

Xperia	No SA	Mild	Moderate	Severe	No SA	Mild	Moderate	Severe
REI < 5	**1**	0	0	0	**0**	0	0	0
5–15	2	**6**	1	0	2	**2**	1	0
15–30	0	1	**5**	1	0	1	**4**	0
REI > 30	0	0	2	**11**	0	0	1	**6**

iPhone	No SA	Mild	Moderate	Severe	No SA	Mild	Moderate	Severe
REI < 5	**3**	0	0	0	**0**	1	0	0
5–13	2	**4**	1	0	3	**1**	0	0
13–36	1	2	**3**	2	0	1	**3**	0
REI > 36	0	0	0	**4**	0	0	0	**3**

*Cutoff values for REI were determined for each device in the training groups and applied to the test groups for that device. The macro F1 scores (mean of the F1 scores for the four severity classes) for Amue Link, Xperia, and iPhone were 0.586, 0.699, and 0.646, respectively, in the training groups and 0.677, 0.788, and 0.714 in the test groups.

The number of correctly classified objects is displayed in bold.

The most common misclassification was the overestimation of SA severity among subjects with no SA. In the training groups, 67% of subjects with no SA were misclassified as having at least mild SA for Amue Link and Xperia, while 50% were misclassified for iPhone. In the test groups, 50%, 100%, and 100% of subjects with no SA were misclassified as having mild or moderate SA for Amue Link, Xperia, and iPhone, respectively.

## Discussion

To our knowledge, this is the first study to demonstrate that SA can be quantitatively detected using only IMUs embedded in non-wristwatch devices, including smartphones. While previous studies have explored the potential of smartphone IMU signals for estimating breathing rates and detecting respiration abnormalities, none have validated their ability to identify SA^33–35^.

Building on our prior findings that IMUs in smartwatches can detect SA^30,31^, the present results suggest that nearly all wearable devices equipped with an IMU could be leveraged for quantitative SA screening. Given that IMUs are already embedded in many consumer devices, this approach has the potential to significantly enhance access to at-home SA screening without requiring specialized medical equipment.

Furthermore, our findings could drive the development of new, low-cost, easy-to-use SA detection devices that operate long-term without frequent recharging, further improving the feasibility of widespread SA monitoring.

Many smartphone applications aim to encourage timely medical care for SA, yet many lack diagnostic accuracy, and their algorithms are often proprietary and unverifiable^24,25^. Some models have shown potential for predicting obstructive SA using smartphone-recorded breathing sounds^26–28^.

For example, Cho et al.^26^ positioned a smartphone 1 m away, applying a neural network model to classify 30-second epochs as “no event,” “apnea,” or “hypopnea” based on spectral features of sound energy. Their model’s REI correlated with PSG AHI (*r* = 0.98) and identified moderate-to-severe SA with an AUC of 0.85, sensitivity of 85%, and specificity of 84%.

The IMU signal-based classification performance in the present study was comparable to those of sound-based methods but offers potential advantages. Unlike sound-based methods, IMU-based detection follows a more straightforward physiological algorithm, can identify all types of SA, including central SA, and is more broadly applicable to various wearable devices.

Two key points should be noted regarding the methods of this study. First, our approach detects respiration using acceleration and gyroscope signals from IMUs attached to the body surface. Since respiratory movement detected from the body surface varies among individuals due to factors such as body shape, nightclothes, bedding, and posture, the detection of apnea and hypopnea needed to be independent of absolute signal levels and amplitudes. To achieve this, we employed a relative reduction in self-ratio—specifically, the percent reduction of the fast envelope from the slow envelope of respiratory amplitude—as a marker of apnea and hypopnea. The effectiveness of this method was validated in a controlled experiment (Appendix Figure S2), where a healthy subject wearing an Amue Link device on the abdomen sequentially assumed different body positions while performing breath-holding. The results demonstrated that breath-hold-induced relative reductions in respiratory amplitude could be detected and that the method remained robust to postural changes.

Second, we detected respiratory movement using an IMU attached to the abdomen, which exhibited a significantly higher signal-to-noise ratio than a smartwatch IMU. The respiratory amplitude measured by the abdominal IMU (acceleration: 10–30 mG, gyroscope: 10–30 dps) was approximately 10 times greater than that recorded by a smartwatch IMU in our previous study (1–3 mG and 1–3 dps, respectively)^30^. While this higher sensitivity improved signal quality, it also made it easier to detect respiratory effort during obstructive apnea, occasionally resulting in the absence of apparent decrease in respiratory amplitude and leading to missed detections of obstructive apnea (Panel A, Fig. 3). To address this, we introduced an auxiliary detection method based on the contrast between respiratory frequency during apnea and that during the resumption of breathing (Panel B, Fig. 3). Since respiratory frequency does not always decrease even when respiratory amplitude drops during apnea or hypopnea, these two methods complement each other effectively.

A strength of this study is that it examined breath-by-breath concordance between REs detected by IMU signals and apnea-hypopnea events detected by PSG. In contrast, previous studies on SA detection using wearable devices ^17–22,26−28^ primarily reported whole-night summary statistics and correlations with AHI, without conducting detailed event-by-event analyses. This limitation raises the possibility that the detected REs may reflect events associated with AHI but distinct from true apnea or hypopnea events, making it difficult to determine the precise applicability and limitations of those methods^29^.

In the present study, REs detected by Amue Link, Xperia, and iPhone demonstrated acceptable breath-by-breath concordance with PSG apnea-hypopnea events, achieving F1 scores of 0.786, 0.821, and 0.796, respectively. Their hourly frequencies also correlated well with PSG-derived AHI (*r* = 0.84, 0.93, and 0.90, respectively).

However, REs were defined as breath cycles containing at least one of four specific events: RAE and RFE derived from acceleration and gyroscope signals. The degree of association between these four events and true apnea-hypopnea events likely varies across event types and IMU devices. This variability highlights why regression models—which incorporate the frequencies of all four events—improved correlations with AHI.

Additionally, REs detected by acceleration and gyroscope sensors may not necessarily be identical. The RAEI derived from acceleration signals was lower than that from gyroscope signals, although the two were closely correlated (Table 3). In contrast, RFEI values from acceleration and gyroscope signals were similar on average, but their correlation varied among devices.

These findings suggest that SA-related respiratory reductions appear in both linear and rotational abdominal movements, but the gyroscope detects reductions in respiratory amplitude more sensitively than the accelerometer. Conversely, reductions in respiratory frequency are, on average, detected equally by both sensors, though relative sensitivity may differ across individuals and devices. Notably, the correlation between acceleration- and gyroscope-derived RFEIs was lower in the Amue Link compared to the Xperia and iPhone. Although this study does not pinpoint the exact cause, device weight may be a contributing factor. The Amue Link, Xperia, and iPhone weigh 23.7 g, 170 g, and 144 g, respectively, and differences in mass could influence vibration characteristics due to inertial forces and variations in contact pressure on the body. These findings support the use of device-specific regression models that incorporate both RAEI and RFEI from acceleration and gyroscope signals to estimate AHI.

Although we observed strong correlations between REIs derived from the regression models and AHI, these correlations could be influenced by the variances of the dependent and independent variables. To further evaluate agreement, we examined the Bland-Altman limits of agreement, which were − 16.7 to 25.9, -17.4 to 22.5, and − 18.4 to 20.5 in the test groups for Amue Link, Xperia, and iPhone, respectively. These limits indicate challenges in distinguishing between subjects with no SA (AHI < 5), mild SA (AHI 5–15), and moderate SA (AHI 15–30). Indeed, the confusion matrix comparing REI-based and AHI-based SA severity classifications revealed substantial misclassification of subjects without SA as having mild SA (Table 7). These findings suggest that the proposed method is best suited for screening moderate-to-severe SA (AHI ≥ 15) or severe SA (AHI ≥ 30), rather than differentiating mild cases from normal respiration.

This study offers significant contributions from both clinical and technological perspectives. Despite its serious health^3–5^ and social^5,8^ implications, over 80% of SA patients remain undiagnosed and untreated^9^. Many individuals fail to seek medical attention unless they experience severe symptoms, and even basic screening devices for SA are not widely accessible^16^. Our finding that IMU signals can be used for quantitative SA screening has the potential to greatly improve the accessibility of SA screening at home, especially by leveraging the widespread availability of IMU-embedded wearable devices.

Additionally, the SA detection capability of the IMU could pave the way for the development of inexpensive, easy-to-use devices integrated with the Internet of Things (IoT) to improve sleep quality. For example, positional SA (AHI in the supine position ≥ 2 × AHI in other positions) has been reported in 55 to 61% of patients with obstructive SA^36,37^, and the effectiveness of positional therapies— such as cervical vertebrae support with head tilting, scapula support in the lateral position^38^, and head-of-bed elevation^39–41^ — has also been demonstrated. Continuous SA monitoring through IMU devices linked to IoT-enabled pillows and beds could facilitate AI-driven feedback therapeutic systems, offering real-time adjustments for optimal sleep positioning.

This study has several limitations. First, the algorithm and model were developed using PSG data from patients with suspected SA (pre-test probability of 45–67%). Applying these results to the general population, where the pre-test probability of SA is lower, or to data collected in home environments, may result in reduced sensitivity and positive predictive value. While this study is the first to demonstrate the potential of smartphone IMU signals for detecting SA and assessing its severity, it does not fully explore optimal signal processing techniques or methods for detecting SA episodes. Future studies should aim to optimize these methods to further enhance detection accuracy.

## Conclusions

Acceleration and gyroscope signals from an IMU embedded in a non-wristwatch device can effectively detect SA episodes and estimate SA severity in adults with suspected SA. Building on our previous findings of quantitative SA detection using smartwatch IMUs, this approach offers significant potential to improve the accessibility of home-based SA screening by utilizing widely available IMU-embedded devices.

## Electronic supplementary material

Below is the link to the electronic supplementary material.

Supplementary Material 1

## Data Availability

The data analyzed in this study is available upon reasonable request from the corresponding author.

## References

[1] Benjafield, A. V. et al. Estimation of the global prevalence and burden of obstructive sleep apnoea: a literature-based analysis. *Lancet Respir Med.* **7**, 687–698. 10.1016/s2213-2600(19)30198-5 (2019). 31300334 10.1016/S2213-2600(19)30198-5 PMC7007763

[2] Mangione, C. M. et al. Screening for obstructive sleep apnea in adults: US preventive services task force recommendation statement. *JAMA* **328**, 1945–1950. 10.1001/jama.2022.20304 (2022). 36378202 10.1001/jama.2022.20304

[3] Somers, V. et al. (ed, K.) Sleep apnea and cardiovascular disease: an American heart association/american college of cardiology foundation scientific statement from the American heart association Council for high blood pressure research professional education committee, Council on clinical cardiology, stroke Council, and Council on cardiovascular nursing. In collaboration with the National heart, lung, and blood Institute National center on sleep disorders research (National institutes of Health). *Circulation* **118** 1080–1111 10.1161/CIRCULATIONAHA.107.189375 (2008). 18725495 10.1161/CIRCULATIONAHA.107.189375

[4] Gharibeh, T. & Mehra, R. Obstructive sleep apnea syndrome: natural history, diagnosis, and emerging treatment options. *Nat. Sci. Sleep.* **2**, 233–255. 10.2147/nss.S6844 (2010). 23616712 10.2147/NSS.S6844 PMC3630950

[5] Morsy, N. E. et al. Obstructive sleep apnea: personal, societal, public health, and legal implications. *Rev. Environ. Health*. **34**, 153–169. 10.1515/reveh-2018-0068 (2019). 31085749 10.1515/reveh-2018-0068

[6] Brunetti, V. et al. Sleep and stroke: opening our eyes to current knowledge of a key relationship. *Curr. Neurol. Neurosci. Rep.* **22**, 767–779. 10.1007/s11910-022-01234-2 (2022). 36190654 10.1007/s11910-022-01234-2 PMC9633474

[7] Tregear, S., Reston, J., Schoelles, K. & Phillips, B. Obstructive sleep apnea and risk of motor vehicle crash: systematic review and meta-analysis. *J. Clin. Sleep. Med.* **5**, 573–581 (2009). 20465027 PMC2792976

[8] Léger, D. & Stepnowsky, C. The economic and societal burden of excessive daytime sleepiness in patients with obstructive sleep apnea. *Sleep Med. Rev.* **51**, 101275. 10.1016/j.smrv.2020.101275 (2020). 32169792 10.1016/j.smrv.2020.101275

[9] Peppard, P. E. et al. Increased prevalence of sleep-disordered breathing in adults. *Am. J. Epidemiol.* **177**, 1006–1014. 10.1093/aje/kws342 (2013). 23589584 10.1093/aje/kws342 PMC3639722

[10] Faria, A., Allen, A. H., Fox, N., Ayas, N. & Laher, I. The public health burden of obstructive sleep apnea. *Sleep. Sci.* **14**, 257–265. 10.5935/1984-0063.20200111 (2021). 35186204 10.5935/1984-0063.20200111 PMC8848533

[11] Wickwire, E. M. Value-based sleep and breathing: health economic aspects of obstructive sleep apnea. *Fac. Rev.* **10**, 40. 10.12703/r/10-40 (2021). 34046644 10.12703/r/10-40 PMC8130410

[12] Epstein, L. J. et al. Clinical guideline for the evaluation, management and long-term care of obstructive sleep apnea in adults. *J. Clin. Sleep. Med.* **5**, 263–276 (2009). 19960649 PMC2699173

[13] Westenberg, J. N. et al. Validation of home portable monitoring for the diagnosis of sleep-disordered breathing in adolescents and adults with neuromuscular disorders. *J. Clin. Sleep. Med.* **17**, 1579–1590. 10.5664/jcsm.9254 (2021). 33739260 10.5664/jcsm.9254 PMC8656910

[14] Pires, G. N., Arnardóttir, E. S., Islind, A. S., Leppänen, T. & McNicholas, W. T. Consumer sleep technology for the screening of obstructive sleep apnea and snoring: current status and a protocol for a systematic review and meta-analysis of diagnostic test accuracy. *J. Sleep. Res.* **13819**10.1111/jsr.13819 (2023). e. 10.1111/jsr.13819 36807680

[15] Espinosa, M. A. et al. Advancements in Home-Based devices for detecting obstructive sleep apnea: A comprehensive study. *Sensors* **23**10.3390/s23239512 (2023). 10.3390/s23239512 PMC10708697 38067885

[16] Punjabi, N. M. The epidemiology of adult obstructive sleep apnea. *Proc. Am. Thorac. Soc.* **5**, 136–143. 10.1513/pats.200709-155MG (2008). 18250205 10.1513/pats.200709-155MG PMC2645248

[17] Pires, G. N., Arnardóttir, E. S., Islind, A. S., Leppänen, T. & McNicholas, W. T. Consumer sleep technology for the screening of obstructive sleep apnea and snoring: current status and a protocol for a systematic review and meta-analysis of diagnostic test accuracy. *J. Sleep. Res.* **32**, e13819. 10.1111/jsr.13819 (2023). 36807680 10.1111/jsr.13819

[18] Chen, Y. et al. A Single-Center validation of the accuracy of a Photoplethysmography-Based smartwatch for screening obstructive sleep apnea. *Nat. Sci. Sleep.* **13**, 1533–1544. 10.2147/nss.S323286 (2021). 34557047 10.2147/NSS.S323286 PMC8453177

[19] Kim, M. W., Park, S. H. & Choi, M. S. Diagnostic performance of Photoplethysmography-Based smartwatch for obstructive sleep apnea. *J. Rhinol*. **29**, 155–162. 10.18787/jr.2022.00424 (2022). 39664308 10.18787/jr.2022.00424 PMC11524370

[20] Zhou, G., Zhou, W., Zhang, Y., Zeng, Z. & Zhao, W. Automatic monitoring of obstructive sleep apnea based on multi-modal signals by phone and smartwatch. Annu Int Conf IEEE Eng Med Biol Soc 1–4, (2023). 10.1109/embc40787.2023.10340237 (2023). 10.1109/EMBC40787.2023.10340237 38083356

[21] Zhou, G. et al. Comparison of OPPO watch sleep analyzer and polysomnography for obstructive sleep apnea screening. *Nat. Sci. Sleep.* **16**, 125–141. 10.2147/nss.S438065 (2024). 38348055 10.2147/NSS.S438065 PMC10860396

[22] Cinar Bilge, P. et al. Scanning of obstructive sleep apnea syndrome using Smartwatch: A comparison of smartwatch and polysomnography. *J. Clin. Neurosci.* **119**, 212–219. 10.1016/j.jocn.2023.12.009 (2024). 38141437 10.1016/j.jocn.2023.12.009

[23] Hayano, J. et al. Quantitative detection of sleep apnea with wearable watch device. *PLoS One*. **15**, e0237279. 10.1371/journal.pone.0237279 (2020). 33166293 10.1371/journal.pone.0237279 PMC7652322

[24] Kapur, V. K. et al. Clinical practice guideline for diagnostic testing for adult obstructive sleep apnea: an American academy of sleep medicine clinical practice guideline. *J. Clin. Sleep. Med.* **13**, 479–504. 10.5664/jcsm.6506 (2017). 28162150 10.5664/jcsm.6506 PMC5337595

[25] Baptista, P. M. et al. A systematic review of smartphone applications and devices for obstructive sleep apnea. *Braz J. Otorhinolaryngol.* **88** (Suppl 5), S188–s197. 10.1016/j.bjorl.2022.01.004 (2022). 35210182 10.1016/j.bjorl.2022.01.004 PMC9801062

[26] Cho, S. W. et al. Evaluating prediction models of sleep apnea from Smartphone-Recorded sleep breathing sounds. *JAMA Otolaryngol. Head Neck Surg.* **148**, 515–521. 10.1001/jamaoto.2022.0244 (2022). 35420648 10.1001/jamaoto.2022.0244 PMC9011176

[27] Le, V. L. et al. Real-Time detection of sleep apnea based on breathing sounds and prediction reinforcement using home noises: algorithm development and validation. *J. Med. Internet Res.* **25**, e44818. 10.2196/44818 (2023). 36811943 10.2196/44818 PMC9996414

[28] Han, S. C. et al. In-Home Smartphone-Based prediction of obstructive sleep apnea in conjunction with level 2 home polysomnography. *JAMA Otolaryngol. Head Neck Surg.* **150**, 22–29. 10.1001/jamaoto.2023.3490 (2024). 37971771 10.1001/jamaoto.2023.3490 PMC10654929

[29] Borsky, M., Serwatko, M., Arnardottir, E. S. & Mallett, J. Toward sleep study automation: detection evaluation of Respiratory-Related events. *IEEE J. Biomedical Health Inf.* **26**, 3418–3426. 10.1109/jbhi.2022.3159727 (2022). 10.1109/JBHI.2022.3159727 35294367

[30] Hayano, J., Adachi, M., Sasaki, F. & Yuda, E. Quantitative detection of sleep apnea in adults using inertial measurement unit embedded in wristwatch wearable devices. *Sci. Rep.* **14**, 4050. 10.1038/s41598-024-54817-z (2024). 38374225 10.1038/s41598-024-54817-z PMC10876631

[31] Hayano, J., Adachi, M., Murakami, Y., Sasaki, F. & Yuda, E. Detection of sleep apnea using only inertial measurement unit signals from Apple watch: a pilot-study with machine learning approach. *Sleep. Breath.* **29**, 91. 10.1007/s11325-025-03255-w (2025). 39891814 10.1007/s11325-025-03255-w PMC11787281

[32] Taebi, A., Solar, B. E., Bomar, A. J., Sandler, R. H. & Mansy, H. A. *Recent. Adv. Seismocardiography Vib.* **2**, 64–86, doi:10.3390/vibration2010005 (2019). 10.3390/vibration2010005 PMC8189030 34113791

[33] Bhongade, A., Gupta, R., Gandhi, T. K. & Ap, P. A. Portable Low-Cost Respiration Rate Measurement System for Sleep Apnea Detection. Annu Int Conf IEEE Eng Med Biol Soc 1–5, (2023). 10.1109/embc40787.2023.10340446 (2023). 10.1109/EMBC40787.2023.10340446 38083784

[34] Hernandez, J. E. & Cretu, E. A wireless, real-time respiratory effort and body position monitoring system for sleep. *Biomed. Signal Process. Control*. **61**, 102023. 10.1016/j.bspc.2020.102023 (2020).

[35] Kontaxis, S. et al. An Inertial-Based wearable system for monitoring vital signs during sleep. *Sensors* **24**10.3390/s24134139 (2024). 10.3390/s24134139 PMC11244494 39000917

[36] Richard, W. et al. The role of sleep position in obstructive sleep apnea syndrome. *Eur. Arch. Otorhinolaryngol.* **263**, 946–950. 10.1007/s00405-006-0090-2 (2006). 16802139 10.1007/s00405-006-0090-2

[37] Garg, H., Er, X. Y., Howarth, T. & Heraganahally, S. S. Positional sleep apnea among regional and remote Australian population and simulated positional treatment effects. *Nat. Sci. Sleep.* **12**, 1123–1135. 10.2147/nss.S286403 (2020). 33304112 10.2147/NSS.S286403 PMC7723233

[38] Lee, J. B. et al. Determining optimal sleep position in patients with positional sleep-disordered breathing using response surface analysis. *J. Sleep. Res.* **18**, 26–35. 10.1111/j.1365-2869.2008.00703.x (2009). 19250173 10.1111/j.1365-2869.2008.00703.x

[39] Souza, F., Genta, P. R., de Souza Filho, A. J., Wellman, A. & Lorenzi-Filho, G. The influence of head-of-bed elevation in patients with obstructive sleep apnea. *Sleep. Breath.* **21**, 815–820. 10.1007/s11325-017-1524-3 (2017). 28647854 10.1007/s11325-017-1524-3 PMC5700252

[40] Iannella, G. et al. Head-Of-Bed elevation (HOBE) for improving positional obstructive sleep apnea (POSA): an experimental study. *J. Clin. Med.* **11**10.3390/jcm11195620 (2022). 10.3390/jcm11195620 PMC9571825 36233488

[41] Lee, S. et al. Implementation of head of bed elevation using adjustable bed and its effects on sleep: A pilot randomized trial. *Altern. Ther. Health Med.* (2024). 38758150

