# Supplementary material for: Detection of sleep apnea using smartphone-embedded inertial measurement unit
Source: Sci Rep. 2025 Apr 28;15:14923. doi: 10.1038/s41598-025-99801-3 (PMC12037849; doi:10.1038/s41598-025-99801-3)
Supplement: Supplementary file 1 — Supplementary Material 1 [file 41598_2025_99801_MOESM1_ESM.docx]

**Appendix**

1. Controlled experiment demonstrating the advantage of the method used in this study for extracting respiratory waves from IMU signals (Figure S1).

**Figure S1.** Controlled experiment demonstrating the advantage of the method used in this study for extracting respiratory waves from IMU signals

Acceleration data were collected from a healthy male subject wearing an Amue Link IMU on the abdomen while assuming a left lateral position and breathing normally. In this instance, respiratory movement was most prominently reflected in the Z-axis, as indicated by the trajectories (G and H). Accordingly, the filtered Z-axis signal (D) exhibited the largest amplitude among the three axes (B, C, and D) and showed a clear respiratory peak in the power spectrum (I). In contrast, the respiratory waveform extracted from the combined IMU signal (E) was less distinct (F), and its power spectrum indicated a low signal-to-noise ratio (J).

**In the left-side panels:**

(A) Three-axis acceleration signals detected by the IMU.

(B, C, and D) Band-pass filtered (0.13–0.66 Hz) accelerations in the X-, Y-, and Z-axes, respectively.

(E) Combined (Comb) signal, calculated as the root sum square of the X-, Y-, and Z-axis data.

(F) Band-pass filtered (0.13–0.66 Hz) Comb signal.

**In the right-side panels:**

(G) 3D trajectory pattern constructed from band-pass filtered X-, Y-, and Z-axis signals.

(H) A rotated view of panel G.

(I) FFT power spectrum of the filtered Z-axis signal.

(J) FFT power spectrum of the filtered Comb signal.

In panels G and H, red dashed lines indicate the zero levels of the X-, Y-, and Z-axes.


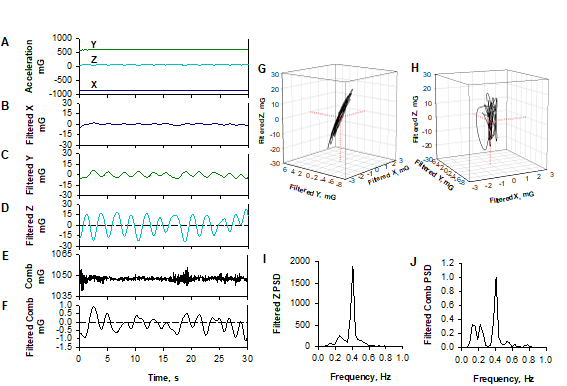


1. Controlled experiment on the effect of breath holding in various body positions on respiratory rate and amplitude measured from IMU acceleration signal using the method in this Study (Figure S2).

**Figure S2.** Controlled experiment on the effect of breath holding in various body positions on respiratory rate and amplitude measured from IMU acceleration signal using the method in this study

Acceleration data were collected from a healthy male subject wearing an Amue Link IMU on the abdomen while sequentially assuming left lateral, supine, right lateral, and prone positions. For each position, data were recorded for one minute. During each position, the subject breathed normally for the first 30 seconds, held their breath for the next 20 seconds (shaded area), and resumed breathing for the final 10 seconds.

From top to bottom:

(A) Three-axis accelerometer signals.

(B, C, and D) Band-pass filtered (0.13–0.66 Hz) accelerations in the X-, Y-, and Z-axes, respectively.

(E) Respiratory signal waveform (RSW), constructed by selecting the axis with the largest respiratory amplitude every 30 seconds from the band-pass filtered X-, Y-, and Z-axis data.

(F) Respiratory frequency (RSF), measured from the RSW using the zero-crossing method.

(G) Respiratory amplitude (RSA), calculated as the root sum square of the band-pass filtered X-, Y-, and Z-axis signals (green line), along with high- and low-frequency upper envelopes (red and blue lines, respectively) for respiratory event detection.


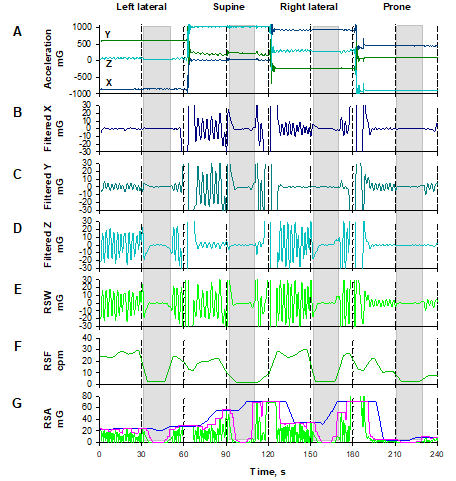


1. Subjects’ characteristics in training and test groups for each device (Table S1). For all devices, there is no significant difference between the training and test groups for any of the parameters.

**Table S1**. Subjects’ characteristics in training and test groups for each device. Data are median (IQR) or frequency (%).

|  | **Amue Link** | | **Xperia** | | **iPhone** | |
| --- | --- | --- | --- | --- | --- | --- |
|  | **Training group** | **Test group** | **Training group** | **Test group** | **Training group** | **Test group** |
| **Number of subjects** | 31 | 15 | 30 | 16 | 22 | 12 |
| **Age, year** | 42 (31-53) | 53 (40-61) | 48 (39-60) | 53 (43-64) | 44 (32-52) | 47 (36-54) |
| **Female, *n* (%)** | 3 (19%) | 3 (10%) | 3 (10%) | 1 (6%) | 6 (25%) | 3 (25%) |
| **BMI, kg/m^2^** | 26.7 (23.4-30.4) | 24.3 (21.6-30.7) | 26.2 (22.5-28.7) | 27.4 (23.8-31.9) | 24.1 (21.6-27.1) | 24.9 (20.9-27.3) |
| ***Polysomnography*** |  |  |  |  |  |  |
| **TRT, min** | 508 (465-527) | 529 (496-557) | 531 (496-554) | 516 (491-553) | 512 (482-545) | 534 (486-565) |
| **TST, min** | 404 (354-435) | 403 (298-445) | 393 (346-431) | 406 (353-424) | 384 (313-438) | 394 (303-445) |
| **Sleep efficiency, %** | 85.7 (79.7-88.5) | 83.9 (75.5-93.5) | 91.9 (78.1-87.7) | 80.9 (70.5-89.2) | 85.2 (79.4-91.1) | 85.6 (77.3-91.9) |
| **Sleep latency, min** | 28.5 (17.5-45.5) | 34.5 (16.0-68.5) | 32.3 (15.0-55.0) | 23.3 (12.5-67.0) | 23.0 (10.5-57.0) | 29.8 (11.8-49.8) |
| **WASO, min** | 28.5 (19.0-44.0) | 32.5 (21.5-49.5) | 43.0 (30.5-95.8) | 40.8 (30.5-57.3) | 37.3 (26.5-56.5) | 30.5 (20.3-64.3) |
| ***Sleep stage*** |  |  |  |  |  |  |
| **N1, %** | 21 (14-31) | 30 (14-35) | 21 (13-34) | 31 (18-41) | 20 (12-32) | 26 (15-32) |
| **N2, %** | 50 (38-60) | 49 (42-52) | 49 (33-54) | 49 (41-59) | 53 (43-62) | 50 (42-53) |
| **N3, %** | 12 (6-21) | 11 (3-21) | 11 (5-23) | 4 (2-7) | 15 (8-22) | 5 (2-18) |
| **REM, %** | 9 (3-16) | 9 (3-19) | 12 (7-19) | 8 (4-20) | 4 (1-16) | 18 (4-23) |
| **Respiratory rate, cpm** | 13.9 (12.6-15.9) | 14.7 (12.9-16.2) | 14.6 (13.9-16.2) | 14.6 (13.7-16.8) | 14.4 (13.1-15.3) | 14.7 (12.6-15.9) |
| **AHI** | 15.8 (8.6-37.4) | 15.4 (8.5-36.7) | 24.0 (10.4-45.0) | 24.4 (9.5-46.7) | 12.0 (5.4-30.2) | 14.7 (6.7-34.7) |
| **OAI** | 3.5 (0.9-8.1) | 4.5 (1.3-7.2) | 7.4 (1.2-18.1) | 5.5 (0.93-13.8) | 3.9 (0.7-5.7) | 2.7 (1.0-7.0) |
| **CAI** | 0.4 (0.2-1.9) | 0.6 (0.2-2.8) | 0.7 (0.1-2.0) | 2.1 (0.2-4.1) | 0.5 (0.2-0.9) | 0.8 (0.4-2.5) |
| **MAI** | 0.3 (0.1-1.8) | 0.2 (0.0-0.8) | 0.4 (0.2-1.8) | 0.9 (0.1-3.2) | 0.2 (0.0-1.0) | 0.5 (0.3-1.3) |
| **HI** | 12.4 (4.3-19.4) | 8.3 (2.1-15.2) | 10.5 (7.4-19.9) | 18.0 (4.4-22.8) | 9.4 (3.5-15.1) | 7.5 (3.9-15.2) |
| **AHI** |  |  |  |  |  |  |
| **< 5** | 6 (19%) | 2 (13%) | 3 (10%) | 2 (13%) | 6 (27%) | 3 (25%) |
| **5-15** | 9 (29%) | 5 (33%) | 7 (23%) | 3 (19%) | 6 (27%) | 3 (25%) |
| **15-30** | 6 (19%) | 3 (21%) | 8 (27%) | 5 (31%) | 4 (19%) | 2 (17%) |
| **≥30** | 10 (33%) | 5 (33%) | 12 (40%) | 6 (37%) | 6 (27%) | 4 (33%) |
| AHI = apnea-hypopnea index, BMI = body mass index, CAI = central apnea index, HI = hypopnea index, MAI = mixed apnea index, OAI = obstructive apnea index, TRT = total recording time, TST = total sleep time, WASO = wake after sleep onset. | | | | | | |

1. Breath-by-breath classification performance for each device across different body positions in the training groups (Table S2). For each body position, each breath was labeled as being either apnea/hypopnea or normal breathing based on polysomnographic judgment and was classified as positive or negative based on respiratory events (REs) defined as a reduction in respiratory amplitude or frequency, derived from acceleration and gyroscope signals.

**Table S2.** Breath-by-breath classification performance for each device across different body positions in the training groups.

| **Device** | **Body Position** | **Number of breathes** | | | | **Classification performance** | | | | |
| --- | --- | --- | --- | --- | --- | --- | --- | --- | --- | --- |
|  |  | **TP** | **FP** | **FN** | **TN** | **Sensitivity** | **Specificity** | **PPV** | **NPV** | **F1 score** |
| **Amue link** | Supine | 1,845 | 274 | 936 | 118,642 | 66.3% | 99.8% | 87.1% | 99.2% | 0.753 |
|  | Lateral | 1,046 | 244 | 298 | 56,692 | 77.8% | 99.6% | 81.1% | 99.5% | 0.794 |
|  | Prone | 22 | 10 | 8 | 3,052 | 73.3% | 99.7% | 68.8% | 99.7% | 0.710 |
| **Xperia** | Supine | 3,002 | 325 | 1,485 | 145,680 | 66.9% | 99.8% | 90.2% | 99.0% | 0.768 |
|  | Lateral | 933 | 125 | 266 | 49,647 | 77.8% | 99.7% | 88.2% | 99.5% | 0.827 |
|  | Prone | 32 | 10 | 17 | 2,759 | 65.3% | 99.6% | 76.2% | 99.4% | 0.703 |
| **iPhone** | Supine | 1,461 | 404 | 790 | 112,504 | 64.9% | 99.6% | 78.3% | 99.3% | 0.710 |
|  | Lateral | 296 | 127 | 137 | 27,703 | 68.4% | 99.5% | 70.0% | 99.5% | 0.692 |
|  | Prone | 1 | 0 | 2 | 343 | - | - | - | - | - |
| TP = true positive, FP = false positive, FN = false negative, TN = true negative, PPV = positive predictive value, NPV = negative predictive value. | | | | | | | | | | |

1. Breath-by-breath classification performance for each device across different body positions in the test groups (Table S3).

**Table S3.** Breath-by-breath classification performance for each device across different body positions in the test groups.

| **Device** | **Body Position** | **Number of breathes** | | | | **Classification performance, %** | | | | |
| --- | --- | --- | --- | --- | --- | --- | --- | --- | --- | --- |
|  |  | **TP** | **FP** | **FN** | **TN** | **Sensitivity** | **Specificity** | **PPV** | **NPV** | **F1 score** |
| **Amue link** | Supine | 1,240 | 278 | 410 | 61,524 | 75.2% | 99.6% | 81.7% | 99.3% | 0.783 |
|  | Lateral | 555 | 151 | 95 | 30,737 | 85.4% | 99.5% | 78.6% | 99.7% | 0.819 |
|  | Prone | 12 | 4 | 17 | 1,886 | 41.4% | 99.8% | 75.0% | 99.1% | 0.533 |
| **Xperia** | Supine | 1,364 | 209 | 417 | 55,612 | 76.6% | 99.6% | 86.7% | 99.3% | 0.813 |
|  | Lateral | 980 | 146 | 212 | 47,591 | 82.2% | 99.7% | 87.0% | 99.6% | 0.846 |
|  | Prone | 13 | 5 | 2 | 1,270 | 86.7% | 99.6% | 72.2% | 99.8% | 0.788 |
| **iPhone** | Supine | 1,156 | 152 | 374 | 57,014 | 75.6% | 99.7% | 88.4% | 99.3% | 0.815 |
|  | Lateral | 124 | 58 | 59 | 14,863 | 67.8% | 99.6% | 68.1% | 99.6% | 0.679 |
|  | Prone | 0 | 0 | 0 | 563 | - | - | - | - | - |
| See the footnote of Table S1 for an explanation of the data. | | | | | | | | | | |
